# Influence of orally fed a select mixture of *Bacillus* probiotics on intestinal T-cell migration in weaned *MUC4* resistant pigs following *Escherichia coli* challenge

**DOI:** 10.1186/s13567-016-0355-8

**Published:** 2016-07-16

**Authors:** Gui-Yan Yang, Yao-Hong Zhu, Wei Zhang, Dong Zhou, Cong-Cong Zhai, Jiu-Feng Wang

**Affiliations:** College of Veterinary Medicine, China Agricultural University, Beijing, 100193 China

## Abstract

Efficient strategies for treating enteritis caused by F4^+^ enterotoxigenic *Escherichia coli* (ETEC)/verocytotoxigenic *Escherichia coli* (VTEC)/enteropathogenic *E. coli* (EPEC) in mucin 4 resistant (*MUC4* RR; supposed to be F4ab/ac receptor–negative [F4ab/acR^−^]) pigs remain elusive. A low (3.9 × 10^8^ CFU/day) or high (7.8 × 10^8^ CFU/day) dose of *Bacillus licheniformis* and *Bacillus subtilis* spore mixture (BLS-mix) was orally administered to *MUC4* RR piglets for 1 week before F4^+^ ETEC/VTEC/EPEC challenge. Orally fed BLS-mix upregulated the expression of TLR4, NOD2, iNOS, IL-8, and IL-22 mRNAs in the small intestine of pigs challenged with *E. coli*. Expression of chemokine CCL28 and its receptor CCR10 mRNAs was upregulated in the jejunum of pigs pretreated with high-dose BLS-mix. Low-dose BLS-mix pretreatment induced an increase in the proportion of peripheral blood CD4^−^CD8^−^ T-cell subpopulations and high-dose BLS-mix induced the expansion of CD4^−^CD8^−^ T cells in the inflamed intestine. Immunostaining revealed that considerable IL-7Rα–expressing cells accumulated at the lamina propria of the inflamed intestines after *E. coli* challenge, even in pigs pretreated with either low- or high-dose BLS-mix, although Western blot analysis of IL-7Rα expression in the intestinal mucosa did not show any change. Our data indicate that oral administration of the probiotic BLS-mix partially ameliorates *E. coli*-induced enteritis through facilitating upregulation of intestinal IL-22 and IκBα expression, and preventing loss of intestinal epithelial barrier integrity via elevating ZO-1 expression. However, IL-22 also elicits an inflammatory response in inflamed intestines as a result of infection with enteropathogenic bacteria.

**Electronic supplementary material:**

The online version of this article (doi:10.1186/s13567-016-0355-8) contains supplementary material, which is available to authorized users.

## Introduction

Enterotoxigenic *Escherichia coli* bearing F4 fimbriae (F4^+^ ETEC) is the most prevalent ETEC strain in causing postweaning diarrhea in pigs [1]. The fimbriae-mediated recognition of specific receptors on host enterocytes is the prerequisite for infection. Breeding programs with F4 receptor–negative pigs is preferable for prevention of F4^+^ ETEC infection, and a polymorphism in the mucin 4 (*MUC4*) gene has been developed to allow genotyping for determining F4ab/ac ETEC resistance/susceptibility [2, 3]. According to this DNA marker-based test, pigs were genotyped as resistant (RR), susceptible heterozygote (SR) and susceptible homozygote (SS). However, *MUC4* RR pigs are now identified not absolutely F4ab/ac receptor–negative (F4ab/acR^−^) pigs, since there are more than 30% showing positive adhesion with F4ab/ac ETEC and more receptors for F4 fimbriae have been discovered [3–5]. We recently found that an F4^+^ enterotoxigenic *E. coli* (ETEC)/verocytotoxigenic *E. coli* (VTEC)/enteropathogenic *E. coli* (EPEC) hybrid can cause enteritis and/or fever in *MUC4* RR pigs. This is possibly due to the ability of this strain to adhere to the intestinal mucosa, and subsequently secrete toxins (e.g. heat-liable, heat-stable enterotoxins, Shiga-like toxin Stx2e) and release LPS [1, 6].

The probiotics *Bacillus licheniformis* and *Bacillus subtilis* are widely used in both humans and animals with a broad spectrum of inhibitory activity against pathogenic bacteria [7, 8]. Our recent study showed that excessive generation of CD4^+^ interleukin (IL)-10–positive T cells following consumption of a *B. licheniformis* and *B. subtilis* mixture (BLS-mix) during episodes of intestinal inflammation caused by F4^+^ ETEC/VTEC/EPEC can inhibit clearance of the pathogen in newly weaned *MUC4* RR pigs [6]. Effective defense against F4^+^ ETEC/VTEC/EPEC achieved through coordination of complex signaling networks linking the innate and adaptive immune systems thus remains elusive.

IL-22 is essential for epithelial defense against extracellular bacteria and critical for mediating mucosal host defenses against attaching and effacing bacteria in the gastrointestinal tract [9]. The central roles of IL-22 in the gut include maintaining normal barrier homeostasis, inducing the secretion of antibacterial proteins, and triggering the expression of chemokines for controlling the spread of invading pathogens [10]. However, IL-22 has both protective and pathologic roles, and the effect of BLS-mix on IL-22 secretion and its role in pigs infected with *E. coli* is poorly understood.

The induction of IL-10–producing Foxp3^−^ T cells by BLS-mix cannot account for the protection of newly weaned *MUC4* RR pigs from F4^+^ ETEC/VTEC/EPEC infection [6]. CD4^+^CD25^+^CD127^low^ cells were used as an alternative marker for regulatory T (Treg) cells, in addition to the conventional CD4^+^CD25^+^Foxp3^+^ population [11]. IL-7 receptor α-chain (IL-7Rα, also known as CD127) contributes to the development of IL-22–producing cells and Treg cells, IL-7/IL-7R–dependent signaling plays a crucial role in regulating the immune response in the intestinal mucosa [12, 13]. In swine, CD127 has been detected in the intestine, lymphoid tissues, and various nonlymphoid tissues [14].

Chemokines can attract specific populations of immune cells to sites of infection or inflammation [15]. Specifically, in humans and mice, the CC chemokine receptor CCR9, expressed by IgA antibody-secreting cells (ASCs) and T cells, responds to its ligand, CCL25, which is selectively expressed in the small intestine and thymus. In contrast, chemokine CCL28, a ligand for CCR10 that is expressed primarily by IgA ASCs and some T lymphocytes, is expressed in mucosa of intestine and elsewhere [16]. In pigs, CCL25 recruits T cells and IgA ASCs that express CCR9 in the gut-associated lymphoid tissues and small intestine, whereas CCL28 can be detected in both intestinal and other mucosal tissues [17]. It remains to be elucidated that the effect of BLS-mix on these two chemokines with their respective receptors in pigs.

Probiotic bacteria increase tight-junction function to modulate the mucosal permeability, but the pathways involved vary depending on the bacterial strain [18, 19]. *Lactobacillus rhamnosus* or *Bifidobacterium lactis* increased the phosphorylation of tight junction proteins zonula occludens-1 (ZO-1) and occludin [20]. Activation of Toll-like receptor 4 (TLR4), nucleotide-binding oligomerization domain 1 (NOD1) and NOD2 by commensal microbiota leads to the degradation of IκBα (the inhibitor of NF-κB), the activation of the transcription factor NF-κB, and release of pro-inflammatory cytokines [21]. Protein kinase C (PKC) has been implicated in regulation of tight junctions in response to luminal bacteria [22].

In the present study, we hypothesized that IL-22 production, T-cell responses, IL-7Rα and tight junction protein in the intestinal mucosa would be involved in the mechanism by which probiotic BLS-mix alleviates the progression of inflammation caused by pathogenic bacteria in newly weaned pigs.

## Materials and methods

### Ethics statement

This study was carried out in strict accordance with the *Guidelines for Laboratory Animal Use and Care* from the Chinese Center for Disease Control and Prevention and in accordance with the *Rules for Medical Laboratory Animals* from the Chinese Ministry of Health, under the protocol (CAU-AEC-2013-073) approved by the Animal Ethics Committee of the China Agricultural University, as described previously [6]. All animals were euthanized under sodium pentobarbital anesthesia, and every effort was made to minimize suffering.

### Animals

A total of 32 *MUC4* RR crossbred (Landrace × Large White) piglets of mixed gender, selected from 8 different litters, weaned at 21 days of age, and weighing 6.80 ± 0.44 kg were obtained from the Beijing Hog Raising and Breeding Center and used in this study. The DNA marker-based test that detection of the polymorphism in gene *MUC4* was used to discriminate resistant (RR) or susceptible (SS, SR) pigs, as described previously [6].

### Bacterial strains

An equal mixture of spray-dried spore-forming *B. licheniformis* (DSM 5749) and *B. subtilis* (DSM 5750) was kindly supplied by Chr. Hansen A/S (Hørsholm, Denmark) and used in this study. The probiotic mixture at a concentration of 3.2 × 10^9^ viable spores/g was resuspended in sterile physiological saline. A low-(3.9 × 10^7^ CFU/mL) or high-dose (7.8 × 10^7^ CFU/mL) solution of the probiotic mixture concentrated in 10 mL of sterile physiological saline was prepared, respectively.

The uncommon *E. coli* strain with serotype/virotype O149:F4ac:LT:STb:Stx2e:eae (named as F4^+^ ETEC/VTEC/EPEC) was obtained from the China Veterinary Culture Collection Center and was grown in Luria–Bertani medium (Oxoid, Basingstoke, UK). Prior to challenge, an inoculum of F4^+^ ETEC/VTEC/EPEC containing 1.0 × 10^9^ CFU/mL resuspended in 10 mL of sterile physiological saline was prepared.

### Experimental design

On the day of weaning (day 0), pigs were assigned to 4 groups (*n* = 8 per group) according to weight and ancestry [6]: control (CONT) group (oral administration of sterile physiological saline); ETEC (oral administration of sterile physiological saline and F4^+^ ETEC/VTEC/EPEC); LDBE (oral administration of low-dose probiotic mixture [3.9 × 10^8^ CFU/day] and F4^+^ ETEC/VTEC/EPEC); and HDBE (oral administration of high-dose probiotic mixture [7.8 × 10^8^ CFU/day] and F4^+^ ETEC/VTEC/EPEC). All animals were maintained by intragastric administration without sedation or gastric acid neutralization.

At 9:00 a.m. on days 1–7, pigs in CONT and ETEC groups were administered with 10 mL of sterile physiological saline orally, while pigs in LDBE and HDBE groups were pretreated with an equal volume of low-dose (3.9 × 10^8^ CFU/day, once daily) or high-dose (7.8 × 10^8^ CFU/day, once daily) BLS-mix solution, respectively. At 9:00 a.m. on day 8, ETEC, LDBE and HDBE pigs were challenged with F4^+^ ETEC/VTEC/EPEC (1.0 × 10^10^ CFU) resuspended in 10 mL of sterile physiological saline orally, whereas CONT pigs received 10 mL of sterile physiological saline only. On day 15 (1 week after F4^+^ ETEC/VTEC/EPEC challenge), pigs in four groups were sacrificed.

### Quantitative PCR amplification of faecal *Escherichia* 16S rRNA gene

To check bacteria shedding, fresh faecal samples were collected from all pigs on days 1, 4, 7, 9 and 12. Genomic DNA was extracted from 200 mg of faeces using a QIAamp DNA Stool Mini Kit (Qiagen, Hilden, Germany) according to the manufacturer’s instructions. Quantitative PCR was performed in ABI 7500 (Applied Biosystems, Foster City, CA, USA). Each reaction mixture (20 μL) contained 1 μL of DNA template, 0.5 μM each primer, and 10 µL of GoTaq qPCR Master Mix (Promega, Madison, WI, USA). The sequences of primers for *Escherichia* used were as follows: 5′-GAGTAAAGTTAATACCTTTGCTCATTG-3′ and 5′-GAGACTCAAGCTKRCCAGTATCAG-3′ [23]. Bacterial DNA standards consisted of serial tenfold dilutions (ranging from 10^0^ to 10^10^ gene copies/μL) of known amounts of purified PCR product obtained from faecal genomic DNA by using specific primers for *Escherichia* 16S rRNA as above. *R*^2^ values for the standard curves were >0.99 and the estimated amplification efficiency was 95–105%. Samples, standards and non-template controls in triplicate were included in each run.

### Flow cytometry

At 0 h (prior to *E. coli* challenge) and 24, 144 h after challenge, 3 mL of peripheral blood from the jugular vein of each pig was collected using Venoject glass tubes (Terumo Europe NV, Leuven, Belgium) containing EDTA. Peripheral blood lymphocytes were isolated by Ficoll gradient centrifugation using Lymphocyte Separation Solution (TBD Science Inc., Tianjin, China), according to the manufacturer’s instructions. In addition, intestinal mucosal lymphocytes [intraepithelial lymphocytes (IELs), lamina propria lymphocytes (LPLs), and Peyer’s patch lymphocytes (PPLs)] from the jejunal and ileal tissue samples of 10-cm in length were isolated as described previously [24].

Briefly, to isolate IELs and LPLs, the opened Peyer’s patches-free tissues were cut into 3-cm pieces. The intestinal pieces were incubated in Hank’s balanced salt solution (HBSS; 10 mM HEPES, 50 U/mL penicillin, and 50 μg/mL streptomycin) containing 3 mM EDTA and 10 mM HEPES and lacking Ca^2+^ and Mg^2+^ (HBSS-EDTA) for 45 min at 37 °C in a shaking incubator, then the solution passed through a sterile 200-μm-pore metal sieve and collected for IELs isolation, the above steps were repeated twice. The remaining tissues were thoroughly washed with RPMI 1640 medium (Gibco, Grand Island, NY, USA), followed by digestion with RPMI-collagenase (RPMI 1640 containing 100 U/mL collagenase VII [Sigma-Aldrich, Saint Louis, MO, USA]) for 45 min at 37 °C in a shaking incubator. After digestion for 3 times, the cell suspension was sedimented at 600 × *g* for 10 min at 4 °C and the resuspended pellet was further purified from the interface between 44 and 66% Percoll gradient (GE Healthcare, Piscataway, NJ, USA). The PPLs were isolated after digestion of the tissues with HBSS-EDTA for 20 min at 37 °C with shaking. The cells were released through gentle mincing and pelleted by centrifugation after passage through a sterile 200-μm metal sieve. Cells were purified and collected from the interface between 40 and 70% Percoll layers.

Cells were counted under a microscope, and viability was tested by trypan blue exclusion. In each reaction, 1 × 10^6^ cells were used for staining and more than 2 × 10^4^ gated events per condition were acquired. Different proportions of these lymphocytes were assessed using CD3/CD4/CD8 triple-color flow cytometry. The following monoclonal antibodies were used: mouse anti-pig CD3ε (clone BB23-8E6-8C8, fluorescein isothiocyanate–conjugated, 559582; BD Biosciences, San Jose, CA, USA), mouse anti-pig CD4α (clone 74-12-4, phycoerythrin-conjugated, 561473; BD Biosciences), and mouse anti-pig CD8α (clone 76-2-11, conjugated to spectral red, GWB-AAEC7C; Gen Way Biotech Inc., San Diego, CA, USA). Data collection was performed using a FACScalibur™ flow cytometer (BD Biosciences). For data analysis, FlowJo software V7.6 (Tree Star) was used.

### Quantitative real-time PCR

Total RNA was extracted from frozen jejunal and ileal tissue samples using Trizol reagent (Invitrogen, Carlsbad, CA, USA) as previously described [25]. The jejunum was sampled without Peyer’s patches involvement. The concentration and purity of the RNA was detected using NanoDrop^®^ ND-2000C spectrophotometer (Thermo Fisher Scientific, Wilmington, DE, USA) and the integrity of RNA was confirmed by agarose gel electrophoresis with ethidium bromide staining and visualization under UV light. Complementary DNA was synthesized from 1 μg of total RNA using the GoScript reverse transcription system (Promega). Transcripts were quantified using SYBR^®^ Premix DimerEraser™ (TakaRa Biotechnology Inc., Dalian, China) on an ABI 7500 Real-time PCR System (Applied Biosystems). To guarantee no genomic DNA contamination, 1 μg of not-reverse-transcribed RNA of each RNA sample was included. The sequences of the primers used are listed in Additional file 1.

Relative mRNA expression was determined by normalization to the geometric mean of the C_T_ values of three selected reference genes, including hypoxanthine phosphoribosyl-transferase (HPRT), glyceraldehyde-3-phosphate dehydrogenase (GAPDH), and β-actin, and the results are presented as fold change, as determined using the 2^−ΔΔCT^ method [6].

### Immunohistochemistry

Proximal, mid, and distal segments (approximately 10 × 15 × 3 mm) of the ileum were fixed in 4% paraformaldehyde, and the samples were then embedded in paraffin and sectioned at 3-μm. The sections were rehydrated, and after antigen retrieval in citrate buffer (10 mM, pH 6), the peroxidase activity of the sections was quenched with 3% H_2_O_2_ in methanol for 30 min. After washing with phosphate-buffered saline (PBS), the sections were blocked with 5% bovine serum albumin in PBS for 30 min and incubated at 4 °C in a humidified chamber for 14 h with polyclonal rabbit anti-human IL-7Rα antibody (ab115249; 1:200 dilution) (Abcam). The sections were labeled with a 1:200 dilution of biotinylated goat anti-rabbit secondary antibody for 2 h at room temperature and then washed three times with PBS. Next, the sections were incubated with StreptAvidin–Biotin Complex (Vectastain Elite ABC Kit; Vector Laboratories, Burlingame, CA, USA) for 1 h at 37 °C. The reaction product was visualized with 3,3′-diaminobenzidine (Zhongshan Golden Bridge Biotechnology Co., Beijing, China). Negative controls were performed using the same procedure with the exception of replacing the primary antibody with PBS and irrelevant rabbit serum in each batch. Images were captured using an Olympus BX41 microscope (Olympus, Tokyo, Japan) equipped with a Canon EOS 550D camera head (Canon, Tokyo, Japan).

### Western blotting

Intestinal tissue samples were collected immediately after animals were euthanized. Each jejunal tissue sample (except for ileal tissues used for IL-7Rα detection) weighing 0.1 g was lysed for 5 min in 1 mL of cold Radio-Immunoprecipitation Assay (RIPA; 50 mM Tris–HCl, pH 8.0, 150 mM sodium chloride, 1.0% Nonidet P-40, 0.5% sodium deoxycholate, 0.1% sodium dodecyl sulfate) buffer supplemented with 5 μL of protease inhibitor cocktail, and 1 mM phenylmethanesulfonyl fluoride (Sigma-Aldrich). The tissue lysates were centrifuged at 12 000 ×* g* for 15 min at 4 °C to remove insoluble material, and the total protein content of the resulting supernatants was quantified using the BCA method (Thermo Fisher Scientific, Waltham, MA, USA). An equal amount of 20 μg of protein extracts from each sample was used for Western blot analyses. The following primary antibodies were used: monoclonal rabbit anti-human IκBα (ab32518, 1:5000 dilution), polyclonal rabbit anti-human IL-7Rα (ab115249, 1:5000 dilution), monoclonal rabbit anti-human PKCα (ab32376, 1:5000 dilution), polyclonal rabbit anti-pig ZO-1 (ab59720, 1:50 dilution), polyclonal rabbit anti-pig occludin (ab31721, 1:250 dilution) (Abcam), monoclonal mouse anti-β-actin (60008-1-Ig, 1:2000 dilution), and monoclonal mouse anti-GAPDH (60004-1-Ig, 1:2000 dilution) (Proteintech, Chicago, IL, USA). The following secondary antibodies used were purchased from Proteintech: horseradish peroxidase–conjugated affinipure goat anti-rabbit IgG (H+L) (SA00001-2) and goat anti-mouse IgG (H+L) (SA00001-1). Immobilon Western chemiluminescent HRP substrate (Millipore, Medford, MA, USA) was used for visualization of the blots. The bands were visualized using Tanon-5200 Gel image system (Tanon, Shanghai, China). The intensity of bands was quantified by densitometric analysis using Image J software (National Institutes of Health, Bethesda, MD, USA). Results are presented as the ratio of the intensity of the IκBα, PKCα, ZO-1, or occludin band to that of the β-actin band or the ratio of the intensity of the IL-7Rα band to that of the GAPDH band.

### Statistical analysis

Statistical analysis was performed using the SAS software package, version 9.3 (SAS Institute Inc., Cary, NC, USA). Data were analyzed using the software’s PROC MIXED procedure, as described previously [6]. The fixed effects of treatment, litter, sex, sampling time, intestinal section, interactions between treatments, and sampling time or intestinal section, as well as random effects associated with individual pigs within a treatment were included in the statistical model. Differences between least-square means were compared using Tukey’s test. Results were presented as the mean ± SEM. A *P* value of <0.05 was considered indicative of statistical significance.

## Results

### BLS-mix consumption reduced the abundance of *Escherichia* in faeces following *E. coli* challenge

Prior to F4^+^ ETEC/VTEC/EPEC challenge, the numbers of *Escherichia* was increased in faeces of LDBE pigs 7 days after BLS-mix administration compared with ETEC pigs (*P* = 0.043; Additional file 2). One day after *E. coli* challenge (day 9), the faeces of ETEC pigs but not pigs pretreated with either low- or high-dose BLS-mix had increased numbers of *Escherichia* compared with CONT pigs (*P* = 0.010). This increase was continuously observed on day 12 (*P* < 0.001).

### Effect of orally fed BLS-mix on TLR4- and NOD-mediated inflammatory responses in the small intestine

**Figure 1:**
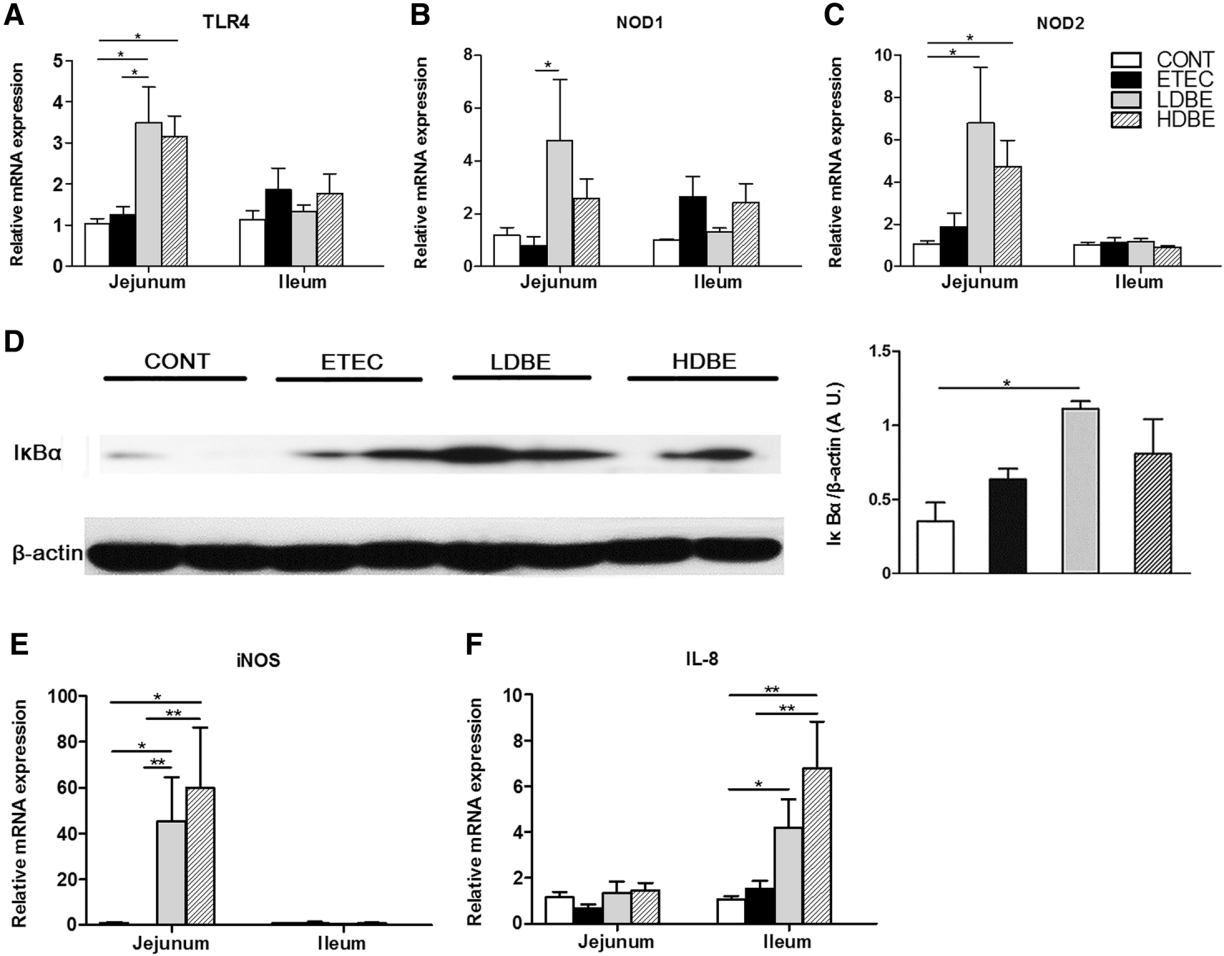
**Effect of orally fed BLS-mix on TLR4- and NOD-mediated inflammatory responses in the small intestine.** The relative expression of mRNAs for genes encoding **A** TLR4, **B** NOD1, **C** NOD2, **E** iNOS, and **F** IL-8 in both jejunal and ileal tissues collected from the indicated pigs 1 week after F4^+^ ETEC/VTEC/EPEC challenge was analyzed using quantitative real-time PCR. **D** Western blot analysis of IκBα expression in jejunal tissues collected from the indicated pigs 1 week after F4^+^ ETEC/VTEC/EPEC challenge. Representative IκBα expression in jejunal tissues collected from 2 pigs of each group is shown (left panel). Results are presented as the ratio of IκBα band intensity to the intensity of the β-actin band (right panel). Data are expressed in arbitrary units (A.U.) as the mean ± SEM for each tissue (*n* = 8 per group). *P < 0.05; **P < 0.01.

The expression of jejunal TLR4 mRNA was higher in pigs pretreated with either low- or high-dose BLS-mix compared with CONT pigs (*P* = 0.014 and *P* = 0.038, respectively), and expression of jejunal TLR4 mRNA was also higher in LDBE pigs than ETEC pigs (*P* = 0.034; Figure 1A). In addition, higher expression of NOD1 mRNA was observed in jejunal tissue of LDBE pigs compared with ETEC pigs (*P* = 0.027; Figure 1B). NOD2 mRNA expression in the jejunal tissues of both LDBE and HDBE pigs was upregulated compared with CONT pigs (*P* = 0.040 and *P* = 0.033, respectively; Figure 1C).

Western blot analysis revealed that the expression of IκBα was higher in the jejunal tissues of LDBE pigs compared with CONT pigs (*P* = 0.011; Figure 1D). Compared with CONT and ETEC pigs, the expression of jejunal iNOS mRNA was upregulated in pigs pretreated with either low- or high-dose BLS-mix (Figure 1E). In addition, the expression of ileal IL-8 mRNA was upregulated in both LDBE and HDBE pigs compared with CONT pigs (*P* = 0.025 and *P* = 0.001, respectively), and it was also higher in HDBE pigs than ETEC pigs (*P* = 0.008; Figure 1F).

### Effect of orally fed BLS-mix on peripheral blood lymphocytes

To investigate whether the immunomodulatory effect associated with BLS-mix involves modulation of systemic lymphocyte subpopulations, we analyzed the CD3^+^ T-cell subpopulations including CD4^+^CD8^−^, CD4^−^CD8^+^, CD4^+^CD8^+^, and CD4^−^CD8^−^ T cells in peripheral blood.

**Figure 2:**
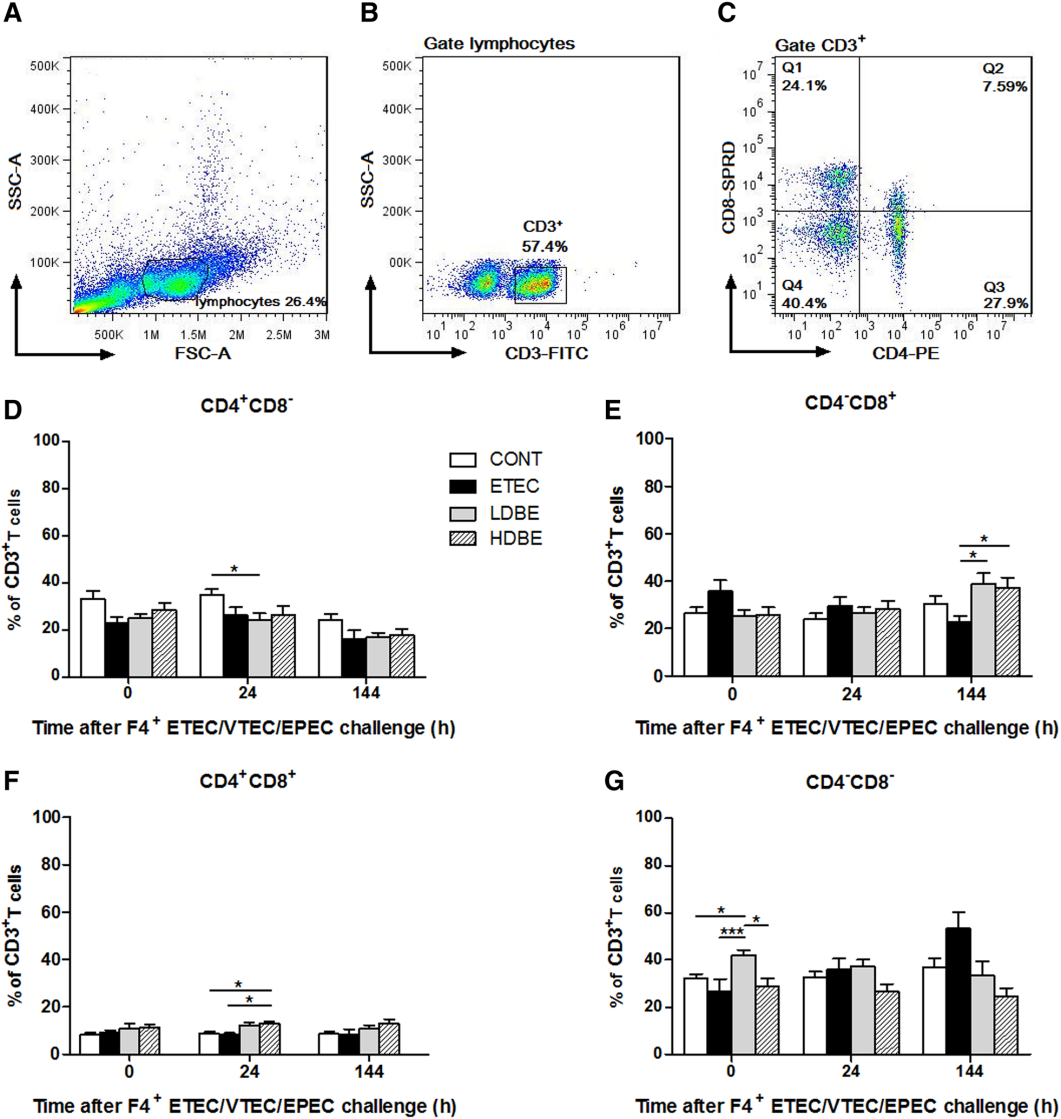
**Effect of orally fed BLS-mix on peripheral blood lymphocytes.** Peripheral blood samples were collected from the indicated pigs at 0, 24, and 144 h after F4^+^ ETEC/VTEC/EPEC challenge. **A** FSC-A/SSC-A dot plot of peripheral blood lymphocytes, cells with no gating. **B** CD3 dot plot, cells were gated on lymphocytes. **C** CD4/CD8 dot plot, cells were gated on CD3^+^. Flow cytometry analysis of the percentages of **D** CD4^+^CD8^−^, **E** CD4^−^CD8^+^, **F** CD4^+^CD8^+^, **G** CD4^−^CD8^−^ cells among CD3^+^ T cells. Data are presented as the mean ± SEM for each time point (*n* = 8 per group). *P < 0.05; ***P < 0.001.

Representative dot plots showed the gating strategy for peripheral blood lymphocytes (Figures 2A–C). Analysis of different T-cell subpopulations revealed that the percentage of CD4^+^CD8^−^ T cells was lower in LDBE pigs than CONT pigs (*P* = 0.048) at 24 h after F4^+^ ETEC/VTEC/EPEC challenge (Figure 2D). In contrary, the percentage of CD4^−^CD8^+^ subpopulation was increased in pigs pretreated with either low- or high-dose BLS-mix compared with ETEC pigs (*P* = 0.021 and *P* = 0.044, respectively) at 144 h after F4^+^ ETEC/VTEC/EPEC challenge (Figure 2E). The percentage of CD4^+^CD8^+^ T cells was higher in HDBE pigs compared with both CONT and ETEC pigs (*P* = 0.030 and *P* = 0.018, respectively) at 24 h after F4^+^ ETEC/VTEC/EPEC challenge (Figure 2F).

Notably, the percentage of CD4^−^CD8^−^ T cells was higher in LDBE but not HDBE pigs than CONT and ETEC pigs 1 week after BLS-mix administration (*P* = 0.035 and *P* < 0.001, respectively). However, the percentage of CD4^−^CD8^−^ T cells did not differ at 24 and 144 h after F4^+^ ETEC/VTEC/EPEC challenge (Figure 2G).

### Effect of orally fed BLS-mix on expression of intestinal IL-22 and selected chemokine mRNAs

The peripheral IL-22 levels in serum were under detection (<5 pg/mL) by ELISA analysis. To explore the role of IL-22 in intestinal mucosa immunity, we quantified the expression of mRNAs for IL-22 and selected chemokines in both jejunal and ileal tissues.

**Figure 3:**
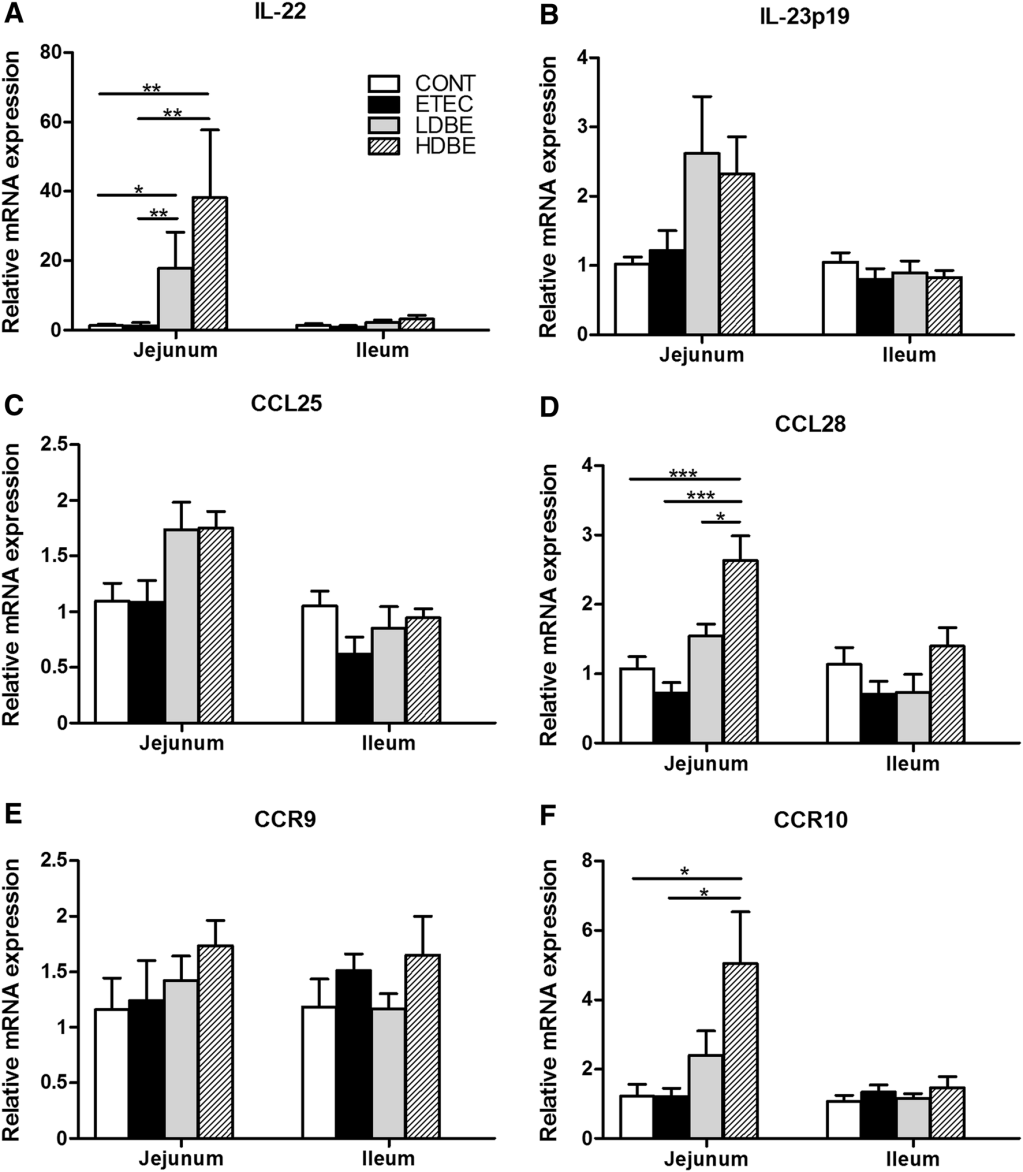
**Orally fed BLS-mix upregulates the expression of IL-22 and chemokine mRNAs in the small intestine.** The expression of mRNAs for the **A** IL-22, **B** IL-23p19, **C** CCL25, **D** CCL28, **E** CCR9, and **F** CCR10 genes in both jejunal and ileal tissues collected from the indicated pigs 1 week after F4^+^ ETEC/VTEC/EPEC challenge was analyzed using quantitative real-time PCR. Data are presented as the mean ± SEM for each tissue (*n* = 8 per group). *P < 0.05; **P < 0.01; ***P < 0.001.

Compared with CONT or ETEC pigs, jejunal IL-22 mRNA expression was upregulated in both LDBE (*P* = 0.040 and *P* = 0.008, respectively) and HDBE (*P* = 0.008 and *P* = 0.002, respectively) pigs (Figure 3A). However, no upregulation of the expression of IL-23p19 mRNA in the small intestine was observed after F4^+^ ETEC/VTEC/EPEC challenge, even in pigs pretreated with BLS-mix (Figure 3B).

Although no changes in the expression of mRNAs for CCL25 and its receptor CCR9 were observed in the small intestine after F4^+^ ETEC/VTEC/EPEC challenge (even in pigs pretreated with BLS-mix), compared with CONT and ETEC pigs, the expression of jejunal CCL28 mRNA was upregulated (*P* < 0.001) in pigs pretreated with high-dose (but not low-dose) BLS-mix (Figures 3C–E). In parallel, jejunal CCR10 mRNA expression was upregulated in pigs pretreated with high-dose (but not low-dose) BLS-mix, compared with CONT and ETEC pigs (*P* = 0.023 and *P* = 0.029, respectively; Figure 3F).

### Presence of IL-7Rα–expressing cells in the ileal mucosa

**Figure 4:**
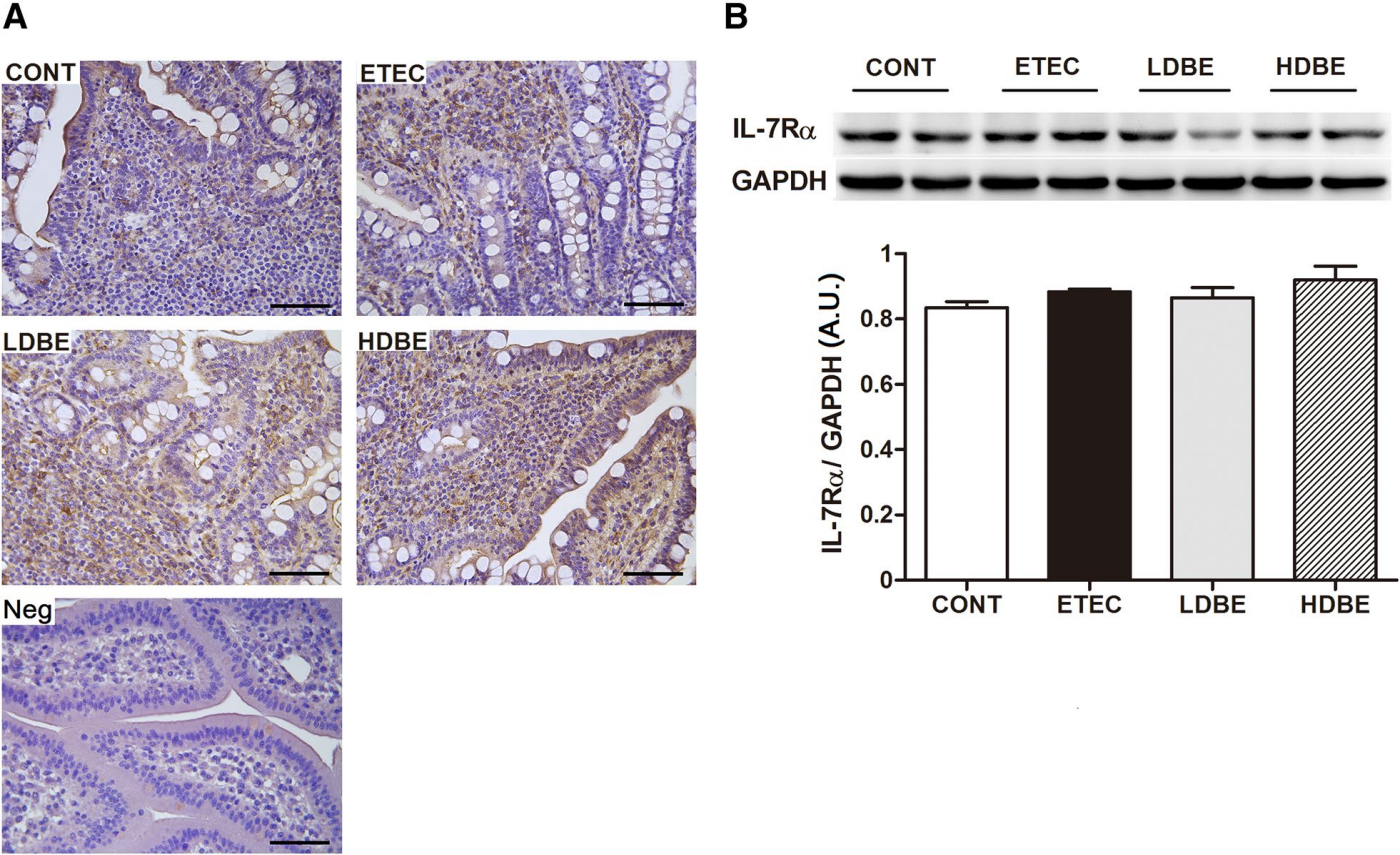
**Presence of IL-7Rα–expressing cells in the ileal mucosa. A** Representative photomicrographs of immunostaining of IL-7Rα and negative control of irrelevant rabbit serum in ileal tissues collected from the indicated pigs 1 week after F4^+^ ETEC/VTEC/EPEC challenge. IL-7Rα–positive cells were predominantly localized in the surface epithelium and the lamina propria, as well as Peyer’s patches, with sporadic positive staining. Scale bars, 100 μm. **B** Representative Western blot results for IL-7Rα in ileal tissues collected from 2 pigs of each group are shown (upper panel). Results are presented as the ratio of the IL-7Rα band intensity to the intensity of the GAPDH band (lower panel). Data are expressed in arbitrary units (A.U.) as the mean ± SEM for each tissue (*n* = 8 per group).

Immunostaining showed that IL-7Rα was predominantly localized in the surface epithelium and the lamina propria, as well as Peyer’s patches, with sporadic positive staining (Figure 4A). A considerable number of IL-7Rα–positive cells infiltrated into the lamina propria of the inflamed mucosa in ETEC pigs, even in those pretreated with either low- or high-dose BLS-mix. However, Western blot analysis revealed no differences in the expression of IL-7Rα among the four groups (Figure 4B).

### Effect of orally fed BLS-mix on intestinal lymphocytes

We assessed changes in different proportions of T-cell subpopulations among lymphocytes in the intestinal compartments, including the Peyer’s patches, the intraepithelial layer, and the lamina propria of the jejunum and ileum.

**Figure 5:**
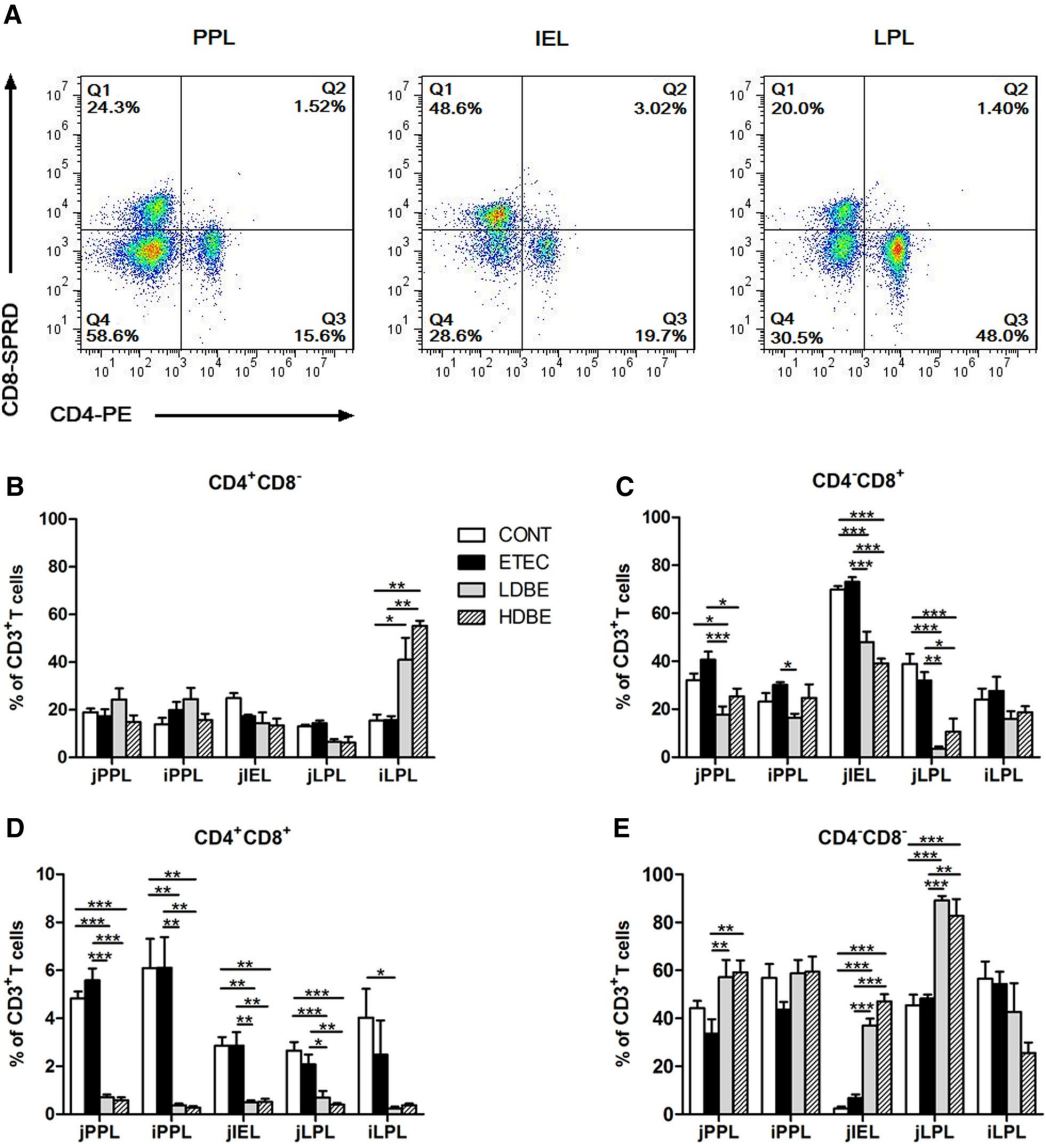
**Effect of orally fed** **BLS-mix on intestinal lymphocytes.** **A** Representative dot plots showed the gating strategy for gut T-cell subpopulations. Flow cytometry analysis of the percentage of **B** CD4^+^CD8^−^, **C** CD4^−^CD8^+^, **D** CD4^+^CD8^+^, **E** CD4^−^CD8^−^ cells among intestinal CD3^+^ T cells. Peyer’s patch lymphocytes (PPLs), intraepithelial lymphocytes (IELs), and lamina propria lymphocytes (LPLs) were collected from jejunal and ileal tissues from the indicated pigs 1 week after F4^+^ ETEC/VTEC/EPEC challenge. Data are presented as the mean ± SEM for each tissue (*n* = 8 per group). *P < 0.05; **P < 0.01; ***P < 0.001.

An increase in the percentage of CD4^+^CD8^−^ T cells among iLPLs was observed in pigs pretreated with either low- or high-dose BLS-mix compared with CONT pigs (*P* = 0.047 and *P* = 0.004, respectively), and this increase in HDBE pigs was also observed compared with ETEC pigs (*P* = 0.003; Figure 5B). However, no changes in the percentage of CD4^+^CD8^−^ T cells among PPLs, jIELs and jLPLs were found after F4^+^ ETEC/VTEC/EPEC challenge, even in pigs pretreated with BLS-mix.

The percentage of CD4^−^CD8^+^ T cells among jPPLs was lower in pigs pretreated with either low- or high-dose BLS-mix than ETEC pigs (*P* < 0.001 and *P* = 0.013, respectively), and this decrease in LDBE pigs was observed compared with CONT pigs (*P* = 0.017; Figure 5C). Similarly, CD4^−^CD8^+^ T cells among jIELs and jLPLs were decreased in pigs pretreated with either a low or high dose of BLS-mix compared with both CONT and ETEC pigs. Similar to subpopulation data, significant decreases in the percentage of CD4^+^CD8^+^ T cells among PPLs, jIELs and jLPLs were observed in pigs pretreated with either low- or high-dose BLS-mix compared with both CONT and ETEC pigs (*P* < 0.05; Figure 5D).

In the jejunum, pigs pretreated with either low- or high-dose BLS-mix had a higher percentage of CD4^−^CD8^−^ T cells in the Peyer’s patches, intraepithelium and lamina propria than did ETEC pigs (*P* < 0.01; Figure 5E). The increased CD4^−^CD8^−^ T cells among jIELs and jLPLs in pigs pretreated with either a low or high dose of BLS-mix were also observed compared with CONT pigs (*P* < 0.001).

### Effect of orally fed BLS-mix on PKC-α, ZO-1, and occludin expression

**Figure 6:**
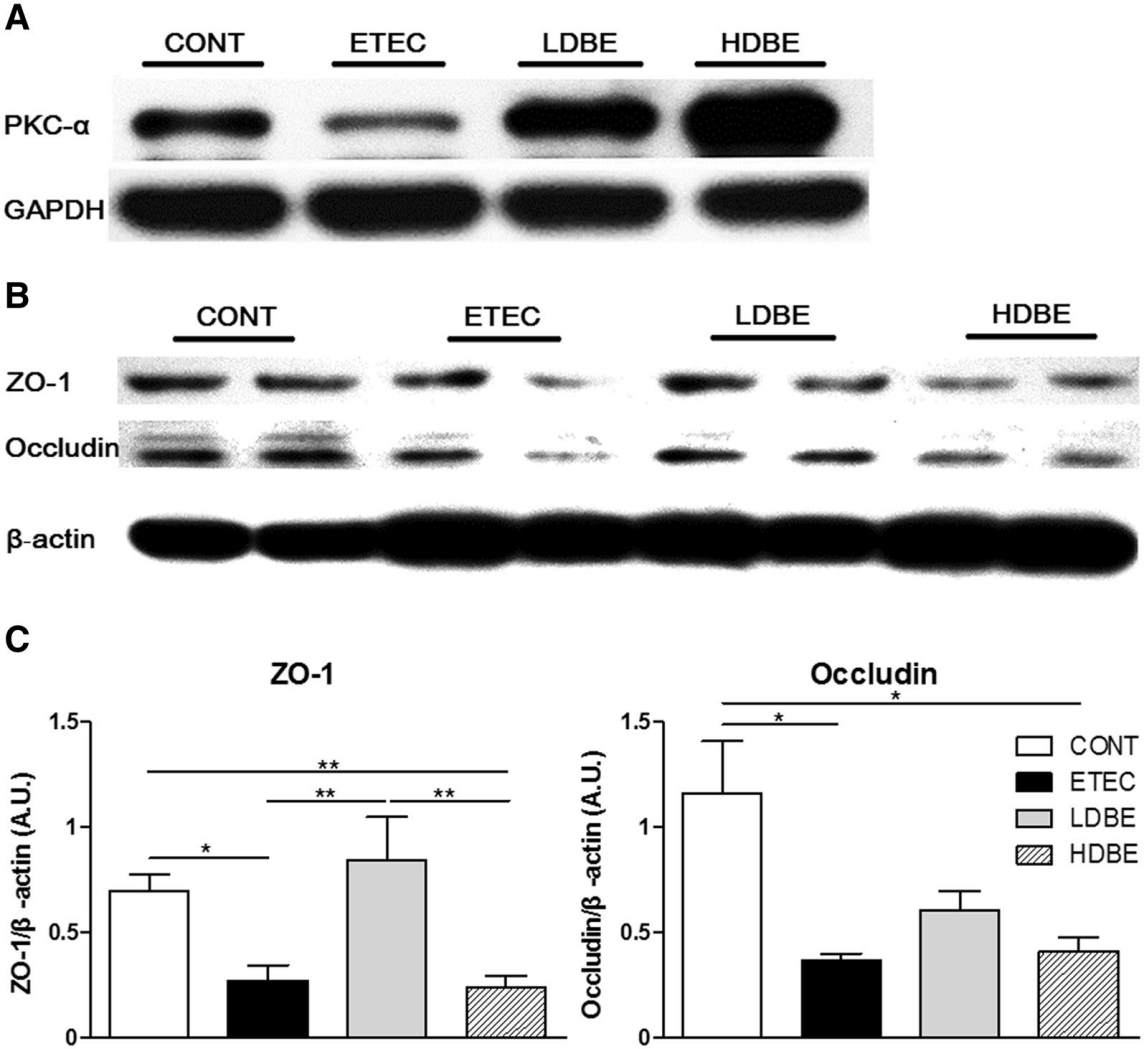
**Effect of orally fed BLS-mix on PKC-α, ZO-1, and occludin expression.** Western blot analysis of PKC-α, zonula occludens-1 (ZO-1), and occludin expression in jejunal tissues collected from pigs 1 week after F4^+^ ETEC/VTEC/EPEC challenge. **A** Representative Western blot results for PKC-α in jejunal tissues collected from 1 pig of each group are shown. **B** Representative Western blot results for ZO-1 and occludin in jejunal tissues collected from 2 pigs of each group are shown. **C** Results are presented as the ratio of the intensity of the ZO-1 or occludin band to the intensity of the β-actin band. Data are expressed in arbitrary units (A.U.) as the mean ± SEM for each tissue (*n* = 8 per group). *P < 0.05; **P < 0.01.

Western blot analysis revealed a reduction in the expression of PKC-α in the jejunum of ETEC pigs compared with CONT pigs (Figure 6A). Consistent with our hypothesis, compared with CONT pigs, the expression of both ZO-1 and occludin was lower in the jejunum of ETEC pigs (but not LDBE pigs) following F4^+^ ETEC/VTEC/EPEC challenge (*P* = 0.013 and *P* = 0.015, respectively; Figures 6B and C). Furthermore, the expression of both ZO-1 and occludin in the jejunum of HDBE pigs was lower compared with CONT pigs (*P* = 0.009 and *P* = 0.020, respectively).

## Discussion

The outcome of F4^+^ ETEC/VTEC/EPEC infection in *MUC4* RR pigs includes fever, anorexia, depression, weight loss, mild diarrhea, the onset of enteritis in the small intestine and excessive systemic inflammatory responses [6]. As more receptors for F4^+^ ETEC have been reported [4], our findings suggest that not only the *MUC4* polymorphism but also one or more of the F4ac receptors should be included in the screening assay for F4ab/acR^−^ pigs.

The use of select probiotic mixtures may allow for tailoring strategies to prevent infectious diseases. Our previous findings indicated that excessive generation of CD4^+^IL-10^+^ T cells induced by oral administration of BLS-mix to newly weaned *MUC4* RR pigs with enteritis caused by an enteric pathogen might prohibit clearance of the pathogen [6]. BLS-mix pretreatment reduces the faecal *Escherichia* shedding following F4^+^ ETEC/VTEC/EPEC challenge. It appears that only low dose of BLS-mix ameliorates *E. coli*-induced enteritis in the jejunum, whereas high dose of BLS-mix makes it worse in both the jejunum and ileum.

Entero-invasive bacteria can directly and indirectly activate NF-κB in IECs, a process that leads to the production of inflammatory mediators such as the pro-inflammatory cytokines IL-1β, IL-6, and TNF-α, the chemokine IL-8, the enzyme iNOS, and various adhesion molecules [26]. In addition to the invading bacteria, the probiotic organism *B. lactis* strain BB12 was also shown to activate NF-κB and trigger a pro-inflammatory response in IECs of germ-free rats after bacterial colonization [27]. Consistently, we found that BLS-mix pretreatment helps to activate NF-κB and induce IL-8 and iNOS mRNA expression, and that increased expression of IκBα might in turn inhibit NF-κB activity in the small intestine of *E. coli*-infected pigs pretreated with low-dose BLS-mix. It has been suggested that activation of the alternative pathway of NF-κB can target genes involved in the secondary lymphoid organs development and adaptive immunity [26, 28].

Moreover, IL-22 induces the activation of NF-κB, leading in turn to increased accumulation of inflammatory mediators [29]. An increased production of IL-22 in the serum was found 1 day after *Salmonella enterica* serovar Enteritidis infection in mice [30]. The systemic IL-22 levels were elevated in Crohn’s disease patients (<24 pg/mL) in comparison with healthy participants and systemic IL-22 should derive from the sites where activated T cells were present [31]. In humans, IL-22–producing CD4^+^ T cells in the peripheral blood are predominantly Th22 cells (approximately 50%), Th1 cells (33%), and Th17 cells (15%) [13]. It has been reported that CD4^−^CD8^−^ T-cell populations composed of γδ T and natural killer T (NKT) cells as well as innate lymphoid cells also produce IL-22 [9, 13]. Flow cytometry analyses of peripheral blood lymphocytes revealed that increases in the proportion of CD4^−^CD8^−^ T-cell subpopulation are induced by oral administration of low-dose BLS-mix, although the level of IL-22 in the serum was extremely low. The number and cytokine profile of γδ T cells which isolated from the peritoneal cavity altered in mice inoculated with pathogens including *E. coli* [32]. In addition to the cell surface–associated IL-22 receptor complex, a soluble IL-22–binding receptor (IL-22–binding protein [IL-22BP]) has been identified, and it has been postulated that an increase in the IL-22:IL-22BP ratio in the inflamed colons is the cause of increased systemic IL-22 levels observed in inflammatory bowel disease [31]. Hence, the BLS-mix–induced expansion of CD4^−^CD8^−^ T cells before an enteric pathogen infection may contribute to the IL-22 production after activation for prevention of the systemic inflammation. The pathologic effects of IL-22 may depend on the uncontrolled release of inflammatory mediators, resulting in inflammatory symptoms. It remains to be determined about the IL-22 secretion ability of peripheral CD4^−^CD8^−^ T cells.

At mucosal surface (e.g. the intestinal tract), IL-22 targets epithelial cells and induces the production of antibacterial proteins and selected chemokines [10]. Consistent with the upregulation of jejunal IL-22 mRNA expression, the expression of jejunal TNF-α, IL-6, and T-bet mRNA is also upregulated in pigs pretreated with low-dose BLS-mix [6]. However, the IL-23p19 mRNA expression in the small intestine was not influenced by BLS-mix pretreatment. It is possible that development of IL-22–producing cells occurs via an IL-6–dependent mechanism and is dependent on the transcription factors T-bet and AhR [33]. IL-22 expression is highly increased in the intestine after infection with *Clostridium difficile* [34]. Although IL-22 is considered to prevent colitis development [35], it also promotes pathogen colonization by suppressing related commensal bacteria in mice [36]. In turn, the gut microbiota and its products are required for the development of IL-22–producing cells and IL-22 production [37]. Our data suggest that IL-22 induced by orally fed BLS-mix not only acts on the intestine to maintain the mucosal homeostasis [9], it also promotes an inflammatory response in inflamed intestinal mucosal tissues as a result of infection with enteropathogenic bacteria. Further research should identify the IL-22–expressing cells in pigs and discover the role of IL-22 in mediating pathogen colonization and intestinal inflammation that caused by enteric pathogens.

The chemokine CCL25 regulate trafficking of T lymphocytes to the gut under physiologic and pathophysiologic conditions [38]. Surprisingly, no changes in the expression of CCL25 mRNA or mRNA of its receptor, CCR9, were observed in the small intestine following F4^+^ ETEC/VTEC/EPEC challenge, even in pigs pretreated with either low- or high-dose BLS-mix. Although CCL25 mRNA expression has been shown to be upregulated in the small intestine in the presence of *E. coli* [39], CCL25 expression is restricted to the small intestine and thymus [16], and CD4^+^Foxp3^+^ Treg cells do not require CCR9 expression to traffic into and function in the inflamed colonic lamina propria in mice [40]. Blockade of CCR9 or CCL25 does not attenuate inflammation during the late stages of chronic murine ileitis [41]. Collectively, CCL25 in the small intestine is considered to play its role in trafficking and homing T cells between bone marrow, lymphoid organs and sites of infection or inflammation for generation of immune responses [38].

Intriguingly, higher levels of mRNA for chemokine CCL28 and its receptor CCR10 were observed in the jejunum of F4^+^ ETEC-infected pigs pretreated with high-dose BLS-mix. A previous study reported upregulation of the expression of CCL28 and CCR10 mRNA in the small intestine of pigs orally inoculated with *E. coli* compared with germ-free pigs [39]. In humans, expression of CCL28 is increased in the inflamed colon of patients with ulcerative colitis [42]. CCL28 production and recruitment of CCR10^+^ Treg cells to the inflamed liver tissue were reported to be involved in the epithelial inflammation in humans [43]. Besides, CCL28 might participate in mediating the accumulation of CCR10-expressing IgA ASCs in the porcine mucosal tissues [44]. Taken together, the upregulated expression of CCL28 and CCR10 in the intestinal mucosa following consumption of high-dose BLS-mix is partly involved in clearance of the pathogen, but also is associated with intestinal inflammation during *E. coli* infection.

The survival of mature and naïve T cells as they circulate between the blood and secondary lymphoid organs requires IL-7/IL-7Rα signaling [12]. However, IL-7 exacerbates chronic colitis, with expansion of mucosal CD4^+^IL-7R^high^ T cells in mice [45]. An increase in the infiltration of IL-7R-positive cells was reported in the lamina propria of TCRα^−/−^ mice with chronic colitis [46]. The proportion of IL-7R-positive macrophages was shown to decrease after treatment with the lysate of the probiotic *Lactobacillus casei* DN-114001 along with LPS, compared with cells treated only with LPS [47]. In the present study, we found that considerable IL-7Rα–expressing cells infiltrate into the lamina propria of inflamed intestinal tissues after F4^+^ ETEC/VTEC/EPEC challenge, even in pigs pretreated with either low- or high-dose BLS-mix. IL-17A–producing IL-7Rα^+^ innate lymphoid cells are potent promoters of intestinal inflammation in *Tbx21*^−*/*−^*Rag2*^−*/*−^ ulcerative colitis mice [48]. Of note, in a recent study of *Citrobacter rodentium* infection in mice, IL-7Rα blockade impaired the bacterial clearance, decreased IL-22 mRNA level, enhanced the structural disruption and intestinal inflammation in colon epithelium, demonstrating that intestinal epithelium-derived IL-7 played a critical role in the protective immunity against intestinal pathogens [49]. The crucial role of IL-7/IL-7Rα signaling in regulating the intestinal inflammation caused by enteric pathogens still needs to be defined.

IL-7Rα is required for the development of γδ T cells and NKT cells in humans and mice [12]. In the present study, not only low-dose BLS-mix consumption induced an increase of CD4^−^CD8^−^ T cells in the jejunum of *E. coli*-infected pigs, but high-dose BLS-mix consumption also enhanced the expansion of CD4^−^CD8^−^ T cells in the inflamed intestine. CD4^−^CD8^−^ αβ and γδ T cells display both pro-inflammatory and regulatory profiles in patients infected with *Mycobacterium tuberculosis* [50]. Resident γδ T cells in mesenteric sites of TCR-β^−*/*−^ mice fuel Th17 responses and actively participate in colitis development, suggesting a pathogenic role of γδ T cells in intestinal inflammation [51]. In addition, as lymph nodes are specialized sites for T-cell priming, effector T cells then migrate from lymph nodes to the sites of infection and inflammation in order to perform their immune functions [15]. The change in the proportion of γδ T cells in Peyer’s patches is probably related with the degree and diversity of gut colonization in swine [52]. Based on these data, we therefore hypothesize that increased CD4^−^CD8^−^ T cells accumulated at the inflamed intestines are implicated in the intestinal inflammation. Further, the subpopulations of CD4^−^CD8^−^ T cells and γδ T cells should be identified in pigs and the interaction of the bacterial colonization with CD4^−^CD8^−^ T-cell response within the inflamed intestine needs to be explored in future investigations.

IL-22–mediated barrier maintenance involves regulating tight junctions between IECs [13]. Tight junction barrier loss is sufficient to cause inflammatory bowel disease [19]. Our results showed that the expression of ZO-1 and occludin decreases after F4^+^ ETEC/VTEC/EPEC infection. Consistent with our results, *E. coli* heat-stable toxin b induces intestinal epithelial barrier dysfunction via altering tight junction proteins [53]. Furthermore,F4^+^*E. coli* infection results in decreased expression of ZO-1 and occludin in porcine intestinal epithelial J2 cells (IPEC-J2) [54]. In accordance with the observed upregulation of IL-22 mRNA expression, pretreatment with the probiotic BLS-mix also enhanced the expression of PKC-α and ZO-1 in jejunal mucosa in the present study. In addition to the probiotic BLS-mix, pretreatment with *L. rhamnosus* also result in upregulation of ZO-1 expression [54]. The biogenesis of tight junctions is regulated selectively by PKC, and it appears that TLR2 enhances ZO-1-associated intestinal epithelial barrier integrity via PKC [22]. Notably, in the present study, administration of low-dose (but not high-dose) BLS-mix promoted expression of the tight junction protein ZO-1. *Bacillus*-derived lipoteichoic acid treatment enhanced the expression of tight junction proteins including ZO-1 and occludin in the IPEC-J2 cells [55]. However, during processes of inflammation, overexpression of inflammatory mediators (e.g. iNOS) can cause cell and tissue damage, and hence leads to the deceased expression of tight junction proteins [56]. Our data indicate that administration of a low dose of probiotic BLS-mix may enhance intestinal epithelial barrier integrity through upregulation of ZO-1 expression; however, inflammatory mediators and changes in PKC-α activity might also contribute to the relocalization of ZO-1 in gut tissues infected with enteropathogenic bacteria.

In conclusion, our data indicate that the development of intestinal inflammation in *MUC4* RR pigs following F4^+^*E. coli* challenge is associated with the accumulation of IL-7Rα–expressing cells in the lamina propria of inflamed intestines. Orally fed probiotic BLS-mix leads to an increase in the expression of intestinal IL-22, IκBα and ZO-1, thereby contributing to the amelioration of intestinal inflammation and maintenance of intestinal epithelial barrier integrity. However, expansion of CD4^−^CD8^−^ T cells and increased IL-22 expression following consumption of BLS-mix may also elicit an inflammatory response involved in intestinal inflammation caused by enteropathogenic bacteria. Our data suggest a low dose (3.9 × 10^8^ CFU/day) of probiotic BLS-mix may allow for protecting the host against enteric pathogens in clinical practice.

## Additional files

10.1186/s13567-016-0355-8 **Information of oligonucleotide primers used for quantitative real-time PCR.** The table shows the sequences of primers used for real-time PCR, length of the respective PCR product and gene accession number in this study.
10.1186/s13567-016-0355-8 **Effect of orally fed BLS-mix on faecal** ***Escherichia*** **shedding before and after** ***E. coli*** **infection.** Fresh faecal samples from animals of the indicated groups were collected on days 1, 4, 7, 9 and 12 after weaning. Bacterial DNA isolated from 200 mg of faeces from pigs among four groups was analyzed by quantitative PCR using universal primers for *Escherichia* 16S rRNA gene. Results are presented as log_10_ copies/g faeces. Data are presented as means ± SEM (*n* = 5 per group). Mean values at the same time point without a common superscript letter differ significantly.

## Abbreviations

BLS-mix: *Bacillus licheniformis* and *Bacillus subtilis* mixture
F4^+^ ETEC: F4 (K88)-positive enterotoxigenic *Escherichia coli*
VTEC: verocytotoxigenic *Escherichia coli*
EPEC: enteropathogenic *Escherichia coli*
*MUC4* RR: mucin 4 resistant
F4ab/acR^−^: F4ab/ac receptor–negative
IEL: intraepithelial lymphocyte
LPL: lamina propria lymphocyte
PPL: Peyer’s patch lymphocyte
Treg: regulatory T cell
CCL: CC-chemokine ligand
CCR: CC-chemokine receptor
IκBα: inhibitor of NF-κB
IL-7Rα: IL-7 receptor α-chain
ZO-1: zonula occludens-1
PKC: protein kinase C
