# Supplementary material for: Influence of orally fed a select mixture of Bacillus probiotics on intestinal T-cell migration in weaned MUC4 resistant pigs following Escherichia coli challenge
Source: Vet Res. 2016 Jul 16;47:71. doi: 10.1186/s13567-016-0355-8 (PMC4947265; doi:10.1186/s13567-016-0355-8)
Supplement: Supplementary file 1 — 10.1186/s13567-016-0355-8 Information of oligonucleotide primers used for quantitative real-time PCR. The table shows the sequences of primers used for real-time PCR, length of the respective PCR product and gene accession number in this study. [file 13567_2016_355_MOESM1_ESM.docx]

**Additional file 1 Information of oligonucleotide primers used for quantitative real-time PCR.**

| **Gene product^a^** | **Primer** | | **Product size (bp)** | **Accession number** | **Reference** |
| --- | --- | --- | --- | --- | --- |
|  | **Direction^b^** | **Sequence (5'→3')** |  |  |  |
| HPRT | F | GTGATAGATCCATTCCTATGACTGTAGA | 104 | U69731 | [[6](#_ENREF_1)] |
|  | R | TGAGAGATCATCTCCACCAATTACTT |  |  |  |
| GAPDH | F | CCAGAACATCATCCCTGCTT | 229 | NM_001206359.1 | [[6](#_ENREF_1)] |
|  | R | GTCCTCAGTGTAGCCCAGGA |  |  |  |
| β-actin | F | CTCTTCCAGCCCTCCTTCCT | 103 | XM_003357928.2 | [[6](#_ENREF_1)] |
|  | R | GCGTAGAGGTCCTCCTGATGT |  |  |  |
| TLR4 | F | GCCATCGCTGCTAACATCATC | 108 | NM_001113039 | [[25](#_ENREF_2)] |
|  | R | CTCATACTCAAAGATACACCATCGG |  |  |  |
| NOD1 | F | ACCGATCCAGTGAGCAGATA | 140 | NM_001114277 | [[25](#_ENREF_2)] |
|  | R | AAGTCCACCAGCTCCATGAT |  |  |  |
| NOD2 | F | GAGCGCATCCTCTTAACTTTCG | 66 | NM_001105295 | [[2](#_ENREF_2)5] |
|  | R | ACGCTCGTGATCCGTGAAC |  |  |  |
| iNOS | F | CTCCAGGTGCCCACGGGAAA | 117 | XM_005669079.1 | This study |
|  | R | TGGGGATACACTCGCCCGCC |  |  |  |
| IL-8 | F | TCCTGCTTTCTGCAGCTCTC | 100 | NM_213867.1 | [[25](#_ENREF_2)] |
|  | R | GGGTGGAAAGGTGTGGAATG |  |  |  |
| IL-22 | F | ATACGGCATTGGCTTAGCTTTT | 143 | XM_001926156.1 | [[57](#_ENREF_3)] |
|  | R | GAGCGCTGCTACCTGGTGA |  |  |  |
| IL-23p19 | F | GAGAAGAGGGAGATGATGAGACTACA | 114 | NM_001130236.1 | [[57](#_ENREF_3)] |
|  | R | GGTGGATCCTTTGCAAGCA |  |  |  |
| CCL25 | F | GCCTACCACAGCCACATTAAG | 136 | NM_001025214.1 | [[39](#_ENREF_4)] |
|  | R | GCTTCCCGCACACCATCTT |  |  |  |
| CCL28 | F | GCTGCTGCACTGAGGTTTC | 145 | NM_001024695.1 | [[39](#_ENREF_4)] |
|  | R | TGAGGGCTGACACAGATTC |  |  |  |
| CCR9 | F | CCAGATGACTACGGCTATGAC | 150 | NM_001001624.1 | [[39](#_ENREF_4)] |
|  | R | GGCACCCACGATGAACAC |  |  |  |
| CCR10 | F | GCCCGCAGAGCAGGTTTCC | 136 | NM_001044563.1 | [[39](#_ENREF_4)] |
|  | R | CAAAGAGACACTGGGTTGGAAG |  |  |  |

**^a^** HPRT = hypoxanthine phosphoribosyl-transferase; GAPDH = glyceraldehyde-3-phosphate dehydrogenase; TLR = Toll-like receptor; NOD = nucleotide-binding oligomerization domain; iNOS = inducible nitric oxide synthase; IL = interleukin; CCL = CC-chemokine ligand; CCR = CC-chemokine receptor.

**^b^** F = forward primer; R = reverse primer.
