# Supplementary figures and images for: Influence of orally fed a select mixture of Bacillus probiotics on intestinal T-cell migration in weaned MUC4 resistant pigs following Escherichia coli challenge
Source: Vet Res. 2016 Jul 16;47:71. doi: 10.1186/s13567-016-0355-8 (PMC4947265; doi:10.1186/s13567-016-0355-8)

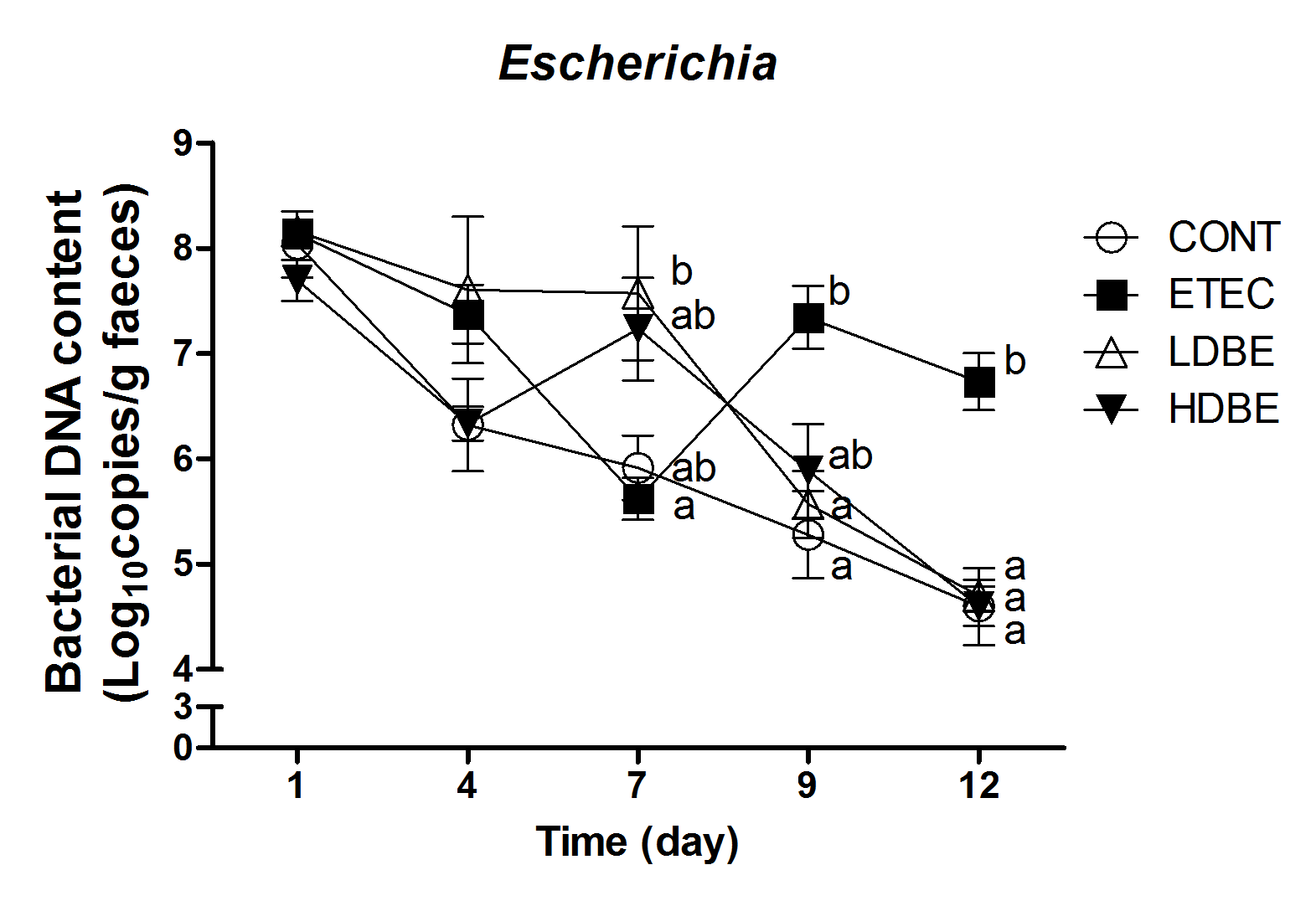

Supplement: Supplementary file 2 — 10.1186/s13567-016-0355-8 Effect of orally fed BLS-mix on faecal Escherichia shedding before and after E. coli infection. Fresh faecal samples from animals of the indicated groups were collected on days 1, 4, 7, 9 and 12 after weaning. Bacterial DNA isolated from 200 mg of faeces from pigs among four groups was analyzed by quantitative PCR using universal primers for Escherichia 16S rRNA gene. Results are presented as log10 copies/g faeces. Data are presented as means ± SEM (n = 5 per group). Mean values at the same time point without a common superscript letter differ significantly. [file 13567_2016_355_MOESM2_ESM.tif]
